# PAVING THE WAY FOR AGE-FRIENDLY UNIVERSITY DESIGNATION: ACCESS AND SUPPORT FOR OLDER ADULT LEARNING INITIATIVES

**DOI:** 10.1093/geroni/igac059.1412

**Published:** 2022-12-20

**Authors:** Katarina Friberg Felsted, Jacqueline Eaton

**Affiliations:** University of Utah, Salt Lake City, Utah, United States; University of Utah College of Nursing, Salt Lake City, Utah, United States

## Abstract

Through a GSA/AARP funded grant, the University of Utah implemented an initiative to promote awareness of a state bill which provides reduced tuition for residents 62 and older. The initiative provided tuition waivers to older adult students, which enhanced enrollment and increased engagement with key age-friendly stakeholders. Implemented over four semesters, student use of tuition waivers increased 875%. This initiative was a crucial step in seeking Age Friendly University (AFU) designation, as it led to mentorship from members of the AFU global network and a partnership with our university’s Emeritus Faculty Board. Strategic planning guided the preparation of a four-step plan towards designation: 1) complete a cross-campus AFU inventory and inform campus stakeholders, 2) report progress to the Council of Deans, 3) present to Academic Vice Presidents, and 4) obtain AFU endorsement from the University President. During this presentation we will present lessons learned as we progress towards designation.

